# Supplementary material for: A Closed Cavity Ultrasonic Resonator Formed by Graphene/PMMA Membrane for Acoustic Application
Source: Micromachines (Basel). 2023 Apr 1;14(4):810. doi: 10.3390/mi14040810 (PMC10143713; doi:10.3390/mi14040810)
Supplement: Supplementary file 1 [file micromachines-14-00810-s001.zip › micromachines-2293076-supplementary.pdf]

Supporting Information: A Closed Cavity Ultrasonic Resonator Formed by Graphene/PMMA Membrane for Acoustic Application

Jing Xu,*,†,‡ Graham. S. Wood,† Enrico Mastropaolo,¶ Peter Lomax,† Michael. J. Newton ,§ and Rebecca Cheung*,†

†*the School of Engineering, Institute for Integrated Micro and Nano Systems, University of Edinburgh, Edinburgh, EH9 3FF, U.K*

‡*AAC Technologies (Scotland) Limited, Edinburgh EH3 8EG, UK*

¶*Deceased 15th July 2019*

§*the Acoustics and Audio Group, University of Edinburgh, Edinburgh, EH8 9DF, U.K.*

E-mail: xujing6@aactechnologies.com; r.cheung@ed.ac.uk

Frequency response of the substrate under mechanical actuation

By applying 1 V AC and 1 V DC to the piezo-disk, the vibration has been observed at the substrate where the graphene/PMMA membrane has been attached, as shown in Fig-ure S1. The resonant frequency of the substrate has been measured to be 169.877 kHz. The side band frequency has been measured to be around 169.446 kHz (Figure 3.a, main text). At the side band frequency (around 169 kHz), the displacement measured at the substrate has

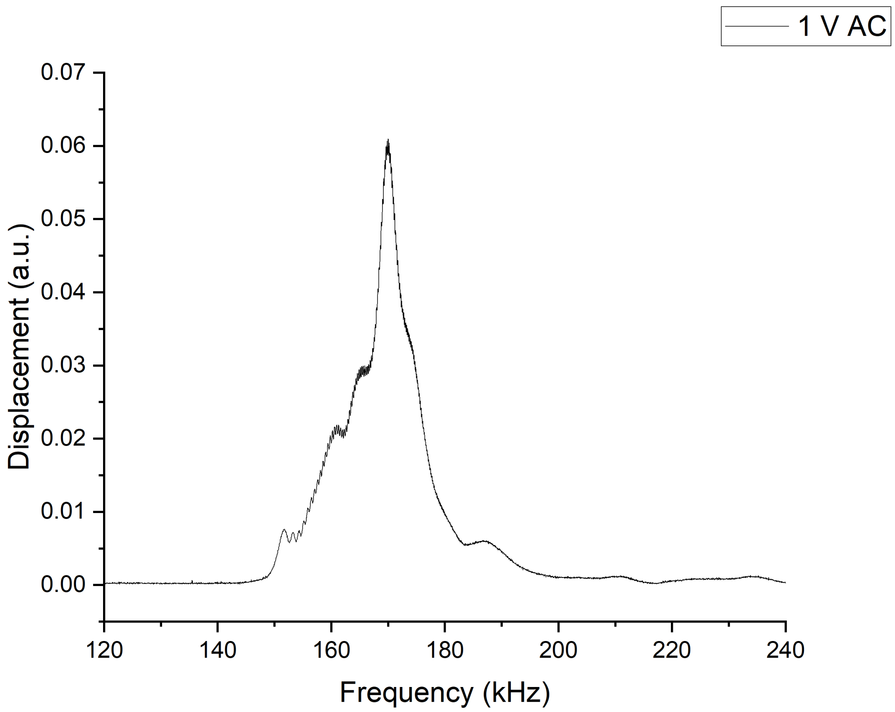

Figure S1: The frequency response of the resonator$'s$ substrate under mechanical actua-tion with the 1 V AC and 1 V DC.

been estimated to be around 12.6 % of the vibration amplitude measured at the suspended membrane.

Transition between (1,1) mode and (0,2) mode under electro-thermal actuation

The mode shape transition between (1, 1) mode and (0, 2) mode has been observed under electro-thermal actuation. As shown in Figure S2, mode shape transition has been meas-ured by applying frequency sweep signal with 7 V AC and 1 V DC between 140 kHz to 220 kHz. The (1, 1) mode (Figure S2.a) has been detected at the frequency of 160.625 kHz. In Figure S2.c, (0, 2) mode has been observed at the frequency of 212.031 kHz. The transition (Figure S2.b) between the (1,1) the (0,2) has been measured at the frequency of 189.063 kHz, which is the likely reason for the side band shown around 188 kHz in Fig-ure 3.c (main text).

Raman spectrum on graphene layer

The Raman spectrum has been measured on the center of the graphene/PMMA membrane using 0.8 mW laser power and with laser excitation of 514.5 nm. As shown in Figure S3, The Raman spectrum has been fitted with Lorentz function. The Raman shift and width of D, G and 2D peaks have been shown in Table S1, which suggests the graphene layer has been transferred onto the closed cavity.[?] The Raman shift of PMMA has been measured at 2958.33 cm^{-1}.[?]

Table S1: Positions, full width at half maximum (FWHM) of the D, G and 2D peaks.

D		G		2D	
position (cm^{-1})	FWHM (cm^{-1})	position (cm^{-1})	FWHM (cm^{-1})	position (cm^{-1})	FWHM (cm^{-1})
1369.9	141.17	1591.5	42.2	2699.6	32.46

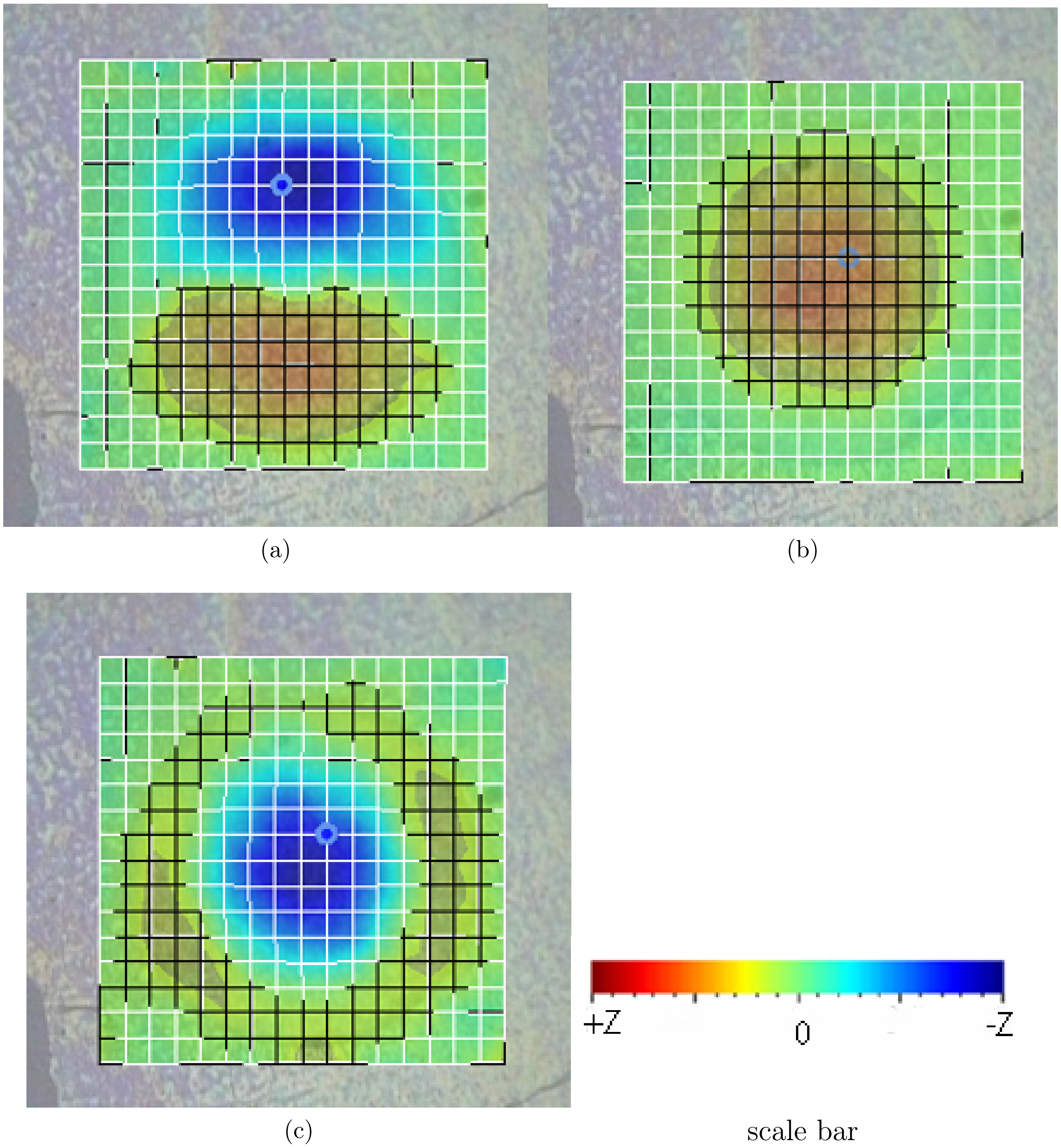

Figure S2: The mode shape of the graphene/PMMA ultrasonic resonator with the electro-thermal actuation (7 V AC, 1 V DC) at the frequency of : (a) 160.625 kHz; (b) 189.063 kHz ; (c) 212.031 kHz.

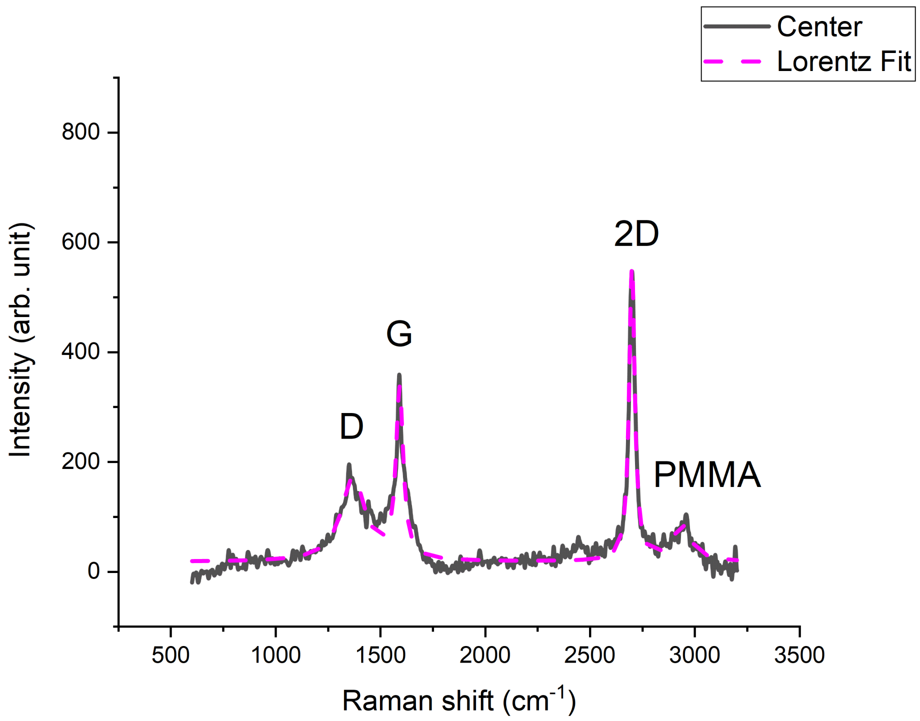

Figure S3: The Raman spectrum of graphene layer suspended over the closed cavity resonator and PMMA peak at $2958.33cm^{-1}$
